# Editorial: Inflammation and heart surgery

**DOI:** 10.3389/fcvm.2024.1493898

**Published:** 2024-09-18

**Authors:** Shahzad G. Raja, Umberto Benedetto, Nandor Marczin

**Affiliations:** ^1^ Department of Cardiac Surgery, Harefield Hospital, London, United Kingdom; ^2^ Department of Cardiac Surgery, University of Studies G. D'Annunzio Chieti and Pescara, Chieti, Italy; ^3^ Department of Anaesthesia and Critical Care, Harefield Hospital, London, United Kingdom; ^4^ Faculty of Medicine, Department of Surgery & Cancer, Imperial College, London, United Kingdom

**Keywords:** cardiac surgery, inflammation, ischaemia and reperfusion injury, oxidative stress, systemic inflammatory response

**Editorial on the Research Topic**
Inflammation and heart surgery

Cardiac surgery involves some of the most intensive and at-risk surgical procedures. Various factors, such as surgical trauma, and the extracorporeal circulation, induce complex systemic inflammatory response as well as organ-specific perioperative morbidity and mortality. Despite advancements in surgical techniques and cardiopulmonary bypass (CPB) technologies over the previous decades, cardiac surgery continues to be associated with these significantly fatal complications (1). An analysis of the Society of Thoracic Surgeons database of >600,000 isolated coronary artery bypass graft procedures identified major postoperative complications including stroke, renal failure, re-operations, and prolonged ventilation in 10.3% patients undergoing this procedure, with a failure to rescue rate in 16%–22% of cases (2).

These complications are further aggravated in other complex procedures and affected by the increasingly aging population presenting for cardiac surgery. The major knowledge gaps in the prevention of these complications as well as monitoring and recovery of these at-risk patients necessitate the need for further research and innovation within these areas.

Various theories regarding the causes of these complications have emerged. The most common of these, identify inflammation, oxidative stress and ischaemia-reperfusion injury as the fundamental mechanisms underlying the pathogenesis of adverse events (3). The contribution of the inflammatory response to post-operative outcomes is increasingly being recognised as an arena for further research.

Tremendous progress has been made in this field; from understanding molecular mechanisms to discovering effective interventions in the laboratory and pre-clinical set up. However, limited data exists on the translation of these insights into everyday clinical practice. We have launched this article collection with an aim to provide a broad overview of the current and emerging research on the role of inflammation in cardiac surgery as well as the definition of best practices in this field.

This special issue on Inflammation and Heart Surgery comprises 11 papers, including 9 original articles, 1 systematic review, and 1 narrative review. The papers cover a broad range of topics focusing on current and emerging research on the role of inflammation in cardiac surgery. The original articles evaluate role of inflammation in perioperative neurocognitive decline, acute kidney injury, endothelial dysfunction, acute type A aortic dissection (ATAAD), mediastinitis, and acute lung injury (ALI) after cardiac surgical interventions. Two original articles evaluate devices while the narrative review gives a comprehensive overview of inflammation after cardiac surgery on cardiopulmonary bypass and strategies to attenuate it. Finally, the systematic review evaluates all currently available infective endocarditis (IE) risk scores to establish effectiveness of existing scores.

Ma et al. tested the hypothesis that the Nod-like receptor family pyrin domain containing 3 (NLRP3) inflammasome, a macromolecular protein complex linked to disorders of the central nervous system, contributes to perioperative neurocognitive decline (PND) and cardiac surgery related PND occurrence is associated with an increase in NLRP3 level. This first in-human study validated the hypothesis and has implications for future research. Krűger et al. in a translational study investigated evolution of wingless-related integration site (WNT) plasma concentration over time and proposed WNT antagonism as a target for further investigation. Yu et al. and Feng et al. complemented existing literature by evaluating role of lymphocyte neutrophil ratio as a predictor of off-pump coronary artery bypass grafting-associated acute kidney injury and neutrophil count as an effective inflammatory index and independent risk factor for in-hospital mortality in patients with ATAAD. On a similar theme, Li et al. make a case for inclusion of interleukin-6 (IL-6) in predictive model for ALI after surgery for thoracic aortic disease. Laudanski et al. investigated impact of cardiac surgery on complement activation and its protective elements (apolipoprotein J and complement factor H) and reported prolonged alterations in complement milieu up to 3 months after cardiac surgery. Risnes et al. investigated the relationship between mediastinitis and troponin T (TnT) and N-terminal pro-brain natriuretic peptide (NT-proBNP) and concluded that raised levels of TnT and NT-proBNP in patients with mediastinitis are surrogates of myocardial injury and cardiac dysfunction. The SedLine device has been recently launched for monitoring of processed electroencephalography (pEEG). Belletti et al. investigated, the previously unknown, impact of temperature on SedLine derived changes in pEEG and provide new insights for clinicians using SedLine for monitoring depth of anesthesia. Geisler et al. evaluated the efficacy of a hemadsorption device, CytoSorb®, and failed to show a significant impact on reduction of IL-6 or periprocedural mortality in patients undergoing complex cardiac surgery. The narrative review by Banerjee et al. provides an expert overview of systemic inflammation after cardiac surgery on CPB and focuses on pharmacological and non-pharmacological strategies to mitigate CPB-related maladaptive inflammatory response. Lastly, the systematic review by Rizzo et al. evaluates all currently available IE scores and concludes that all scores have inherent limitations with lack of external validation, restricting their global utilization.

The spectrum of manuscripts in this special issue clearly suggests that a lot of research is going on in the arena of inflammation and heart surgery in different research avenues, all of them equally exhilarating and pertinent. However, they also highlight that we are at crossroads advancing the theory, expanding the translational evaluation, and leveraging fundamental basic science into clinical patient benefit.

The spectrum of basic science or translational research manuscripts in this collection demonstrates some exciting progress but we are still far from a comprehensive understanding of the principle dilemmas. There is no doubt, that all patients experience a degree of systemic inflammatory response with oxidative stress, leukocyte, and mediator activation but we still have not identified the global transcriptomic, proteomic and metabolic shifts that can differentiate mild and transient responses from those contributing significantly to postoperative complications. Similarly, we do not have a clear handle of patients’ perioperative phenotypes that underpin susceptibility and predisposition of some patients for a higher inflammatory response with pathogenic contribution. While a multimodality approach is advocated targeting multiple steps in the inflammatory response, such an effort will only be successful if the component contribution is relevant.

Thus, in the era of global genetics, personal phenotyping, unparallelled bioinformatics repertoire, and artificial intelligence, there is an urgent need to fully uncover the precise interactions between genetic predisposition and the multiple environmental exposure modulating the patients' biochemical and immunological responses to perioperative trauma and operative procedural factors in causing clinical harm in high risk patients. We are of the opinion that this hugely important topic has not yet mobilised the greatest partnership between basic scientists and clinicians at national and international levels to place systemic inflammatory response syndrome (SIRS) on top of the agenda of surgical sciences. We advocate bringing together the leading force of basic scientists in the field of oxidative stress, immunology, and inflammation and clinical academics covering the full perioperative spectrum of cardiac surgery to fully define what needs to be done to decode the mechanisms, mediators, effector pathways, and the most sophisticated means to target these for clinical benefit. This now needs leading international co-operation between academia, clinicians, and industry under the auspices of major international professional societies.

Lots of work has been done and we are at the crossroads of SIRS research. This special issue collates some of these research activities focusing on new research lines and potential therapeutic targets emphasising the need for international consensus development.
